# Expert Perspectives on the Performance of Explosive Detection Canines: Performance Degrading Factors

**DOI:** 10.3390/ani11071978

**Published:** 2021-07-01

**Authors:** Brian D. Farr, Cynthia M. Otto, Julia E. Szymczak

**Affiliations:** 1 Army Medical Department Student Detachment, 187th Medical Battalion, 32nd Medical Brigade, Joint Base San Antonio-Fort Sam Houston, San Antonio, TX 78006, USA; 2 Penn Vet Working Dog Center, Clinical Sciences and Advanced Medicine, School of Veterinary Medicine, University of Pennsylvania, Philadelphia, PA 19019, USA; cmotto@vet.upenn.edu; 3 Department of Biostatistics, Epidemiology and Informatics, Perelman School of Medicine, University of Pennsylvania, Philadelphia, PA 19019, USA; jszymcza@pennmedicine.upenn.edu

**Keywords:** explosive, detection, canine, performance, requirements, deployment

## Abstract

**Simple Summary:**

Explosive detection canines are a unique resource used to protect a peaceful way of life. Searching for explosives is a difficult task that exposes both the canine and their handler to many factors that may affect their performance. Understanding these factors is essential to measuring and supporting the performance of the explosive detection canine team. This study is the first to systematically document these factors and uses expert interviews to learn from the handlers, trainers, and leaders closest to explosive detection canines. Through these interviews, numerous factors were identified in the areas of how the canine is utilized, the canine–handler interaction, and the physical, climate, operational, and explosive odor environments. Many of these factors are related to how the canine is used, a portion are known before the search starts, and some are only revealed during the search. This organized understanding of the challenges explosive detection canines face enables enhanced selection, training, assessment, and utilization and research into performance sustainment.

**Abstract:**

The explosive detection canine (EDC) team is currently the best available mobile sensor capability in the fight against explosive threats. While the EDC can perform at a high level, the EDC team faces numerous factors during the search process that may degrade performance. Understanding these factors is key to effective selection, training, assessment, deployment, and operationalizable research. A systematic description of these factors is absent from the literature. This qualitative study leveraged the perspectives of expert EDC handlers, trainers, and leaders (*n* = 17) to determine the factors that degrade EDC performance. The participants revealed factors specific to utilization, the EDC team, and the physical, climate, operational, and explosive odor environments. Key results were the reality of performance degradation, the impact of the handler, and the importance of preparation. This study’s results can help improve EDC selection, training, assessment, and deployment and further research into sustaining EDC performance.

## 1. Introduction

The value of explosive detection canines (EDCs) in neutralizing and deterring threats to public safety is well understood [1,2,3,4,5]. We have detailed the requirements these EDCs must meet while performing their lifesaving functions [6]. The EDC team is unique compared to other explosive detection methods, as mammals on both ends of the leash must perform to the same high level for successful detection. The performance of the EDC and handler is dynamic, and searching for explosives in operational settings exposes the team to additional factors, some of which may be unidentified at the start of the search, that may affect the team’s performance [7,8].

Successful EDC selection, training, assessment, utilization, and research rely on a detailed understanding of both the operational requirements and the factors degrading operational performance [8,9,10]. While some of these factors have been documented and explored in the literature, our knowledge remains incomplete. These performance-degrading factors are best understood by EDC practitioners: the handlers, trainers, and leadership who have experienced their effects in operational settings [11,12,13]. The institutional knowledge these individuals have accumulated, especially during the global war on terrorism, is a precious resource that must be systematically captured to benefit current and future EDCs. Much of this institutional knowledge is informally collected and shared amongst EDC practitioners during training, and this oral tradition means important information may be limited to current and recent practitioners [13,14]. A broad and methodical capture of this information is necessary, while remaining cognizant of the challenges with documenting critical information and accumulating and analyzing unclassified information [6,15].

We sought to identify the perceptions that EDC practitioners have of factors degrading EDC performance. Our goal was to recruit highly experienced individuals from across the spectrum of EDC-utilizing organizations to reveal both the factors common to EDCs and those unique to specific utilizations. In our analysis, we aimed to generate hypotheses for the causes and effects of each factor, setting the stage for future research.

## 2. Materials and Methods

### 2.1. Study Design and Participants

In-depth, semi-structured interviews with EDC experts from four utilization sectors (law enforcement, military, federal, and private) were conducted. These sectors were selected as they represent the primary domains in which EDCs operate. This qualitative study was led by a working dog veterinary practitioner (BDF) with expertise in EDC care, training, and program management and graduate training in qualitative research, in collaboration with a working dog veterinary practitioner (CMO) with expertise in detection dog research, and a medical sociologist (JES) with expertise in mixed-methods research. We sought to create a purposive sample that included individuals in each EDC utilization sector with a broad range of experience as a handler, trainer, and/or leader. We limited the sample to U.S.-based EDC practitioners for this study. Subject matter experts were identified through key individuals at representative organizations that employ, train, or manage EDC teams for each utilization sector. These experts were recruited by email, and no incentive was offered for participation. The protocol was deemed exempt by the University of Pennsylvania Institutional Review Board.

### 2.2. Data Collection

Semi-structured interviews were conducted from April to May 2020. The interview guide contained open-ended questions intended to elicit participant knowledge and perceptions of operational requirements of EDCs (see Supplementary Materials for the guide). The key domains in the guide were the participant’s experience with EDC team performance and the physical, climate, operational, and explosive odor environments. One interview was conducted in person, and the remainder were conducted via telephone by BDF. Prior to the start of the interview, participants were made aware of this study’s purpose to characterize the operational performance requirements of EDCs. The interviewer assured potential participants that any sensitive or classified information shared during the interview would be redacted from the final dataset and no personally identifiable information would be collected. With permission of the participant, the interview was audio recorded.

### 2.3. Data Analysis

All audio files were transcribed and uploaded into NVivo software (version 12, QSR International, Doncaster, Australia) for coding [16]. Data were analyzed using a flexible coding approach by one coder (BDF) in conjunction with two collaborators [17]. The interview guide was reviewed to guide the development of an index codebook, which was applied by BDF to two transcripts. The study team reviewed the application of the codebook to the data and suggested revisions. Discrepancies were resolved by consensus. Once the final codebook was developed, it was applied, line by line, to all transcripts. Once all transcripts were coded, we examined patterns of themes within key domains across interviews. We summarized repeated themes across respondents and grouped them into two distinct categories: operational requirements of EDCs and performance-degrading factors. The results we report in this manuscript are themes that were endorsed by the majority of participants.

### 2.4. Security Review and Omitted Data

The analyzed data were reviewed by the appropriate Department of Defense security offices and by individuals experienced in EDC operations. Any data determined to be inappropriate for publication were omitted.

## 3. Results

### 3.1. Characteristics of Study Participants

Interviews were conducted with 17 EDC experts. Over half (*n* = 11, 64.7%) of the participants had experience as an EDC handler, and nearly all had experience as an EDC trainer (*n* = 14, 82.4%) or in leadership over EDC teams (*n* = 14, 82.4%). The majority of participants had experienced employment in multiple occupational settings, with 52.9% (*n* = 9) having experience in the military, 29.4% (*n* = 5) in law enforcement, 23.5% (*n* = 4) in the federal sector and 70.6% (*n* = 12) in the private sector. Participants with experience as an EDC handler, trainer, or leader had an average of 8.0 (range 1–17), 18.2 (range 2–35), and 17.6 (range 2–25) years of experience, respectively. Participants with an experience as an EDC handler or trainer had experience in these roles an average of 14.3 (range 0–28) and 5.4 (range 0–23) years ago, respectively, and all participants with experience as a leader were currently in that role. Many of the participants had additional experience selecting and procuring potential EDCs, certifying and auditing EDCs and EDC teams, consulting, teaching, participating in EDC working groups, and EDC-related research, development, testing, and evaluation. Interviews ranged in length from 36 to 74 min, with a median of 58 min. In the sections that follow, exemplar quotes to support key themes will be provided in the accompanying tables.

### 3.2. The Context of EDC Utilization: Degrees of Control

As we have previously reported, participants in this study described the variety of ways in which EDCs are generally used for screening and/or clearing operations, including the operational environment in which they are expected to perform [6]. In considering the factors that shape performance, our participants suggested it was important to recognize that EDC operations involve varying degrees of control over the situation—high, moderate, and low. Highly controlled operations include indoor clearance operations for dignitaries, demining operations, or building clearance in response to a bomb threat. In these operations, the area to be cleared is usually empty, and the clearance is performed with little or no time pressure (Table 1, Quote 1). Operations with moderate control include clearing active warehouses or large maritime vessels, screening crowds, or military cordon and search operations. In these operations the lower control means the EDC will experience a more complex situation with increased stimuli and more time pressure (Table 1, Quote 2). Some operations are innately difficult to control. An EDC clearing the exterior of a building prior to a military or law enforcement raid or the interior of a building during the raid is subject to a highly complex situation with significant stimuli and time pressure that may affect its performance (Table 1, Quote 3). EDCs also performing criminal apprehension or assault tasks face additional challenges, especially if these additional roles occur immediately prior to or concurrent with detection (Table 1, Quote 4).

### 3.3. EDC Team Factors—Handler Behavior

Participants stated that to sustain high levels of performance, the EDC and handler must function well together. This team performance starts with handler skill and orientation to the work (Table 2, Quote 5). Participants explained that handler frustration with the EDC or decreased interest in the work may cause handlers to reduce their reward frequency, correct unwanted behavior too aggressively, or shift to more compulsion-based handling techniques (Table 2, Quote 6). Handler interactions may result in the EDC becoming too handler-dependent and unwilling to work away from the handler appropriately (Table 2, Quote 7). Participants suggested that handlers required to conduct repeated searches at the same location often experience a malaise in which they may not interact with the EDC as frequently or in their usual manner, demotivating the EDC and leading to a reduction in the thoroughness of the search. Conversely, handlers with a high suspicion of encountering an explosive may be too involved in the search process and not let the EDC work independently (Table 2, Quote 8).

Given the variable and dynamic environments in which they work, handlers must be able to anticipate a change in EDC performance and take the appropriate steps to restore the EDC to an effective level. Handlers who do not sufficiently understand the detection process, lack sufficient rapport or experience with the EDC, or do not understand the EDC’s particular searching abilities may not respond appropriately. These handlers may miss the signs of altered performance or pull the EDC away from explosive odor by failing to recognize the appropriate cues (Table 2, Quote 9).

Participants explained that EDCs may perform at a reduced level when they do not interact with their handler appropriately. An EDC that is too dependent on the handler may try to appease the handler, look for cues from the handler, or be unwilling to search away from the handler (Table 2, Quote 10). On the other end of the spectrum, an EDC that is too independent from the handler may not respond to direction to search productive or missed areas (Table 2, Quote 11). EDCs may also break the search pattern and move around the search area randomly. This dependency or excessive independence may be a result of either inappropriate actions by the handler or an immature EDC-handler bond.

### 3.4. EDC Team Factors—Physical Limitations of the EDC

Participants stated that, in addition to handler behavior and skill, EDCs performance may be degraded by their own physical limitations. Hunger, thirst, pent-up energy from lack of activity, or a need to urinate or defecate may affect their performance. EDCs with insufficient athleticism may be unable to access and traverse elevated, unstable, or restricted spaces. A lack of climate acclimatization, olfactory endurance, or physical fitness (e.g., cardiopulmonary and muscular endurance) may cause an EDC to alter its search behavior or miss an explosive odor (Table 3, Quote 12). Discomfort or pain from illness or a musculoskeletal or neurological issue may cause an EDC to avoid movements (e.g., standing on the hind limbs) necessary for effective performance (Table 3, Quote 13).

### 3.5. Physical Environmental Factors

#### 3.5.1. Manmade Physical Environment Factors

Participants described multiple manmade physical environment factors that can influence the performance of EDCs. Specifically, performance may be degraded by novel surfaces, confined spaces, elevated spaces, novel equipment, and lighting, described in detail below.

##### Manmade Surfaces

EDCs are commonly adversely affected by slippery, transparent, rough, sharp, moving, or unstable surfaces. Transitions between surface types, even if the EDC is familiar with both surfaces, may affect performance. Negative effects range from minor distraction to an inability to locate the source of explosive odor (Table 4, Quote 14).

Some EDCs may encounter novel surfaces during operational detection. These dogs may have limited or no time to acclimate to these surfaces. A highly controlled clearance operation (e.g., for a dignitary) may allow a handler to observe hesitation or distraction in their EDC and adjust accordingly, whereas, in a less controlled clearance operation (e.g., during a low-light assault), reduced performance may go unnoticed by the handler. An EDC performing detection out of sight of their handler may refuse to enter an area with a novel surface leaving that area unsearched but presumed cleared, potentially resulting in harm to personnel.

EDCs may struggle on novel surfaces due to fear, anxiety, distraction, and lack of exposure during critical developmental periods. The novel or uncomfortable physical stimuli may distract them from the challenging mental task of explosive detection.

##### Confined Spaces

EDCs may be required to search confined spaces (e.g., restrooms or cockpit of an aircraft) or pass through them to access other areas (e.g., tunnel systems or narrow spaces running the length of an enclosed commercial trailer). Confined spaces adversely affect performance either due to the EDC being unwilling to enter them or being unable to turn around and exit headfirst (Table 4, Quote 15). EDCs may demonstrate an unwillingness to search these areas, stress responses during the search, or an unwillingness to respond (if their trained final response is a sit or down) near the source of the odor. Participants suggested that organizations utilizing EDCs to search confined spaces may elect to employ EDCs of smaller size to mitigate some of these factors. As with novel surfaces, an insufficiently acclimated EDC encountering a confined space during operational detection may leave an area uncleared to the detriment of the personnel accompanying them (Table 4, Quote 16).

Degraded performance in confined spaces may result from fear, distraction, lack of exposure, inadequate training, and insufficient neuromuscular fitness. The EDC handler may also not be familiar with the effect of confined spaces on their EDC’s behavior.

##### Elevated Areas

EDCs experience degraded performance when searching the top of a building and abruptly obtaining perspective on their elevation by seeing through either the floor or walls (Table 4, Quote 17). An EDC may be affected by this elevation either situationally (e.g., hesitant to search near an edge it can see through or on a transparent surface) or chronologically (e.g., a decrease in performance after becoming aware of the elevation).

##### Novel or Noxious Equipment

EDCs may exhibit degraded performance near novel or noxious equipment. The top floors of industrial and commercial buildings or entertainment and sporting venues often contain communication, electrical, plumbing, and heating, ventilation, and air conditioning equipment. These areas are potentially also confined, accessed by elevated catwalks with metal grate flooring, and dark, further complicating the challenge (Table 4, Quote 18). EDCs may encounter unfamiliar machinery, sudden or loud sounds, noxious or harmful gases, distracting odors, or heat (Table 4, Quote 19).

Equipment of this kind may affect EDC performance due to a lack of prior acclimation or encountering unfamiliar equipment. The accumulation of visual, tactile, and auditory stimuli may be sufficiently distracting to affect performance.

##### Light

Unless appropriately acclimated, EDCs may be afraid of or hesitant to go into low or no-light areas. Detection performance may also drop off immediately after a rapid transition in lighting (Table 4, Quote 20). While canine vision is more adept in low-light situations than human vision, the rapid reduction in visual capacity or overwhelming increase in the light available for dark-adapted eyes may limit navigational abilities or distract from the detection task.

##### Manmade Airflow

EDCs depend on odor capture while searching. Odor near a large vehicle may be pushed away with the vehicle’s arrival, and odor away from the vehicle may be pulled toward the former location of the vehicle as it departs. Ventilation systems may move explosive odor from the source to a different location inside or outside the structure or from outside the structure to the inside. Similarly, open exterior windows and doors or fans at floor level and higher disrupt airflow and may challenge an EDC. The airflow inside a structure is altered by walls, opened or closed doors, furniture, hallways, stairwells, and elevators. The airflow outside a structure is disrupted as the air encounters the structure. These factors may cause an EDC to miss the presence of explosive odor entirely, be unable to locate the source of the odor, falsely indicate due to stress or frustration, or indicate in a location where odor has been concentrated but that is not the location of the explosive (Table 4, Quote 21).

#### 3.5.2. Natural Physical Environment Factors

Surfaces, subterranean areas, and airflow in the natural physical environment may degrade EDC performance. While subterranean areas may only be encountered by certain EDCs, all EDCs must deal with the surfaces and wind patterns of the natural physical environment

##### Natural Surfaces

As with manmade surfaces, any novel natural surface may degrade the performance of an EDC. Encountering novel natural surfaces is unusual for most EDCs, but EDCs deployed to geographically distant locations may encounter and be expected to perform on unfamiliar surfaces. EDCs may be affected by gravel, rocky surfaces, noxious vegetation, and sand. These surfaces may cause immediate discomfort, distraction, and unwillingness to enter or remain in an environment, and traversing sand may result in minor soft tissue injuries to the distal limbs, and lead to future degraded performance (Table 5, Quote 22).

##### Subterranean Areas

Whether natural, modified by humans, or entirely manmade, subterranean areas challenge EDCs (Table 5, Quote 23). Some subterranean areas are poorly lit, confined, and have inclined, novel, or uncomfortable surfaces. Subterranean areas may contain noxious or toxic gases an EDC will encounter before associated personnel and that could affect their olfactory system. The airflow in these areas may be static, keeping explosive odor close to the source, or unpredictable, affecting an EDC’s ability to locate the source. EDCs working off-leash or on a long line may quickly move out of sight of their handler making communication and recognition of responses to odor or noxious stimuli difficult.

##### Natural Airflow

Similar to airflow inside or around manmade structures, natural terrain, terrain modified by humans, and vegetation alter airflow in the natural environment. Tall vegetation, ditches, potholes, vehicle ruts, and currently empty seasonal bodies of water may create areas with no airflow. Whether performing the precision work of searching for landmines or doing more rapid air scenting while moving along a route, an EDC must either independently navigate its nose to these areas or be directed to do so by its handler (Table 5, Quote 24). Failure by both EDC and handler to perform these functions may result in an explosive being missed and harm being inflicted on the EDC or accompanying personnel.

### 3.6. Climate Environmental Factors

Heat and humidity, wind, and exposure to novel situations without acclimatization may degrade an EDC’s performance directly and through its effects on the handler. The altered demeanor of a handler resulting from physical discomfort in environmental extremes may degrade the EDC’s performance (Table 6, Quote 25). Heat (e.g., the ambient temperature, direct exposure to sunlight, and hot surfaces) and humidity may significantly affect an EDC’s performance (Table 6, Quote 26). An elevated ambient temperature increases the burden of thermoregulation on an EDC already working to respire while navigating the physical environment and sniffing for explosives. Elevated humidity decreases the effectiveness of the EDC’s natural evaporative cooling mechanisms, and exposure to sunlight directly heats the EDC through radiation. These three factors may limit the EDC’s ability to effectively sniff with a closed mouth, thereby decreasing detection performance, or they may require the EDC to reduce their work-to-rest ratio. These factors in addition to hot surfaces may also distract the EDC from its detection task. EDCs working in hot or humid environments without the benefit of appropriate acclimatization may perform at an even more degraded level (Table 6, Quote 27).

EDCs may be unable to detect an explosive odor due to the wind altering the path of the molecules, and if they do detect explosive odor, they may be unable to identify the source. EDCs who have not been previously exposed to novel climates (e.g., snow) or asked to detect in more common climates (e.g., rain) may be distracted or uncomfortable, resulting in reduced performance (Table 6, Quote 28).

### 3.7. Operational Environmental Factors

Each EDC may experience many or just a few operational environment factors depending on their individual organization and utilization. These factors may also affect the EDC handler (especially if personal safety is at risk) and limit their ability to direct or monitor their EDC.

An EDC may respond to the behavior and emotion of their handler and surrounding personnel. These individuals may be excited and anxious (e.g., in preparation for a raid or when explosives are expected to be found), or they may be depressed and bored (e.g., when repeatedly searching an area) (Table 2, Quote 8). The EDC itself may show decreased hunt drive when asked to search an area again after a short interval (Table 7, Quote 29). EDCs may be highly distracted by the sight or smell of other animals. Smaller animals may be viewed as a food source, similarly sized or larger animals may be viewed as a companion or a threat, and intact females may present an opportunity to reproduce (Table 7, Quote 30). People in the environment may be a degrading factor. People may distract the EDC by their presence (e.g., nearby security personnel), novelty (e.g., affiliated personnel unknown to the EDC), positive interactions (e.g., giving physical or verbal reinforcement), negative interactions (e.g., direct prolonged eye contact), novel behavior (e.g., yelling, running, or intoxication), or novel presentation (e.g., detained, injured, or deceased) (Table 7, Quote 31). Exposure to radically different background odors without appropriate acclimation or operating around food odor (especially for food reward EDCs) may also degrade performance (Table 7, Quote 32).

EDCs may be affected by sudden (e.g., fireworks), noxious (e.g., loud or high-pitched), or novel (e.g., children screaming) sounds, or sounds associated with positive (e.g., human voices) or stimulating (e.g., gunfire or similar sounds for a criminal apprehension or assault EDC) experiences (Table 7, Quote 33). Prolonged transport or confinement in a novel vehicle may degrade performance upon arrival to a search location (Table 7, Quote 34). EDCs working away from the handler (e.g., on a long line, off-leash, or out of sight) may be more difficult for the handler to direct and monitor for changes in behavior or performance. Finally, EDCs trained to engage people may be distracted from the search task by visual (e.g., observing a potential target), behavioral (e.g., actions by the handler or affiliated personnel), or audible (e.g., gunfire, sirens, or yelling) cues indicating an engagement is imminent (Table 7, Quote 35). Similarly, an EDC who recently performed an engagement may have their performance degraded upon resuming detection. Each of these operational environment factors may cause distraction and unpredictable performance and impair the ability to recover or rapidly acclimate. The EDC may also become fatigued from indirectly experiencing these factors before being asked to perform detection (Table 7, Quote 36).

### 3.8. Explosive Odor Environmental Factors

Target odor availability, location, and type, and the effects of climate and terrain may degrade the performance of the EDC. A small amount of explosive with a low surface area presents a smaller scent picture to the EDC. EDCs with a detection threshold above what is required for that scent picture may miss small amounts of odor (Table 8, Quote 37). In addition, a subtle change in behavior associated with an operational explosive may be missed by handlers who are used to the response their EDC displays when responding to larger scent pictures (Table 8, Quote 38).

Any attempt to conceal the odor decreases the number of molecules available for detection, and masking the odor (e.g., intentionally with distractor or noxious odors or unintentionally with background odors) may degrade the EDC’s ability to identify the target odor (Table 8, Quote 39). Background odors similar to those found in explosives (e.g., petroleum fuels) may cause an EDC with an insufficient ability to discriminate to falsely respond. Repeated false responses may degrade the trust the handler and supporting personnel place in the EDC. Each explosive has a different vapor pressure that affects the rate at which molecules become available for detection (Table 8, Quote 40). The time interval between placement of the explosive and attempted detection by the EDC also plays a role. An explosive with a low vapor pressure that was placed shortly before the EDC searches that area may be more challenging to locate (Table 8, Quote 41). The ability of EDCs to detect explosives may be degraded by placement in atypical locations (e.g., buried, within a wall, or far off the ground) or in objects in which the EDC is not experienced in identifying explosives. In each of these situations, the EDC may be unable to identify the source of the odor and may be unsure of the appropriate response. An explosive odor encountered operationally may differ (e.g., homemade explosive with different ingredients or standard explosive with regional differences) from what the EDC experienced in training, and an insufficient ability to generalize may degrade performance (Table 8, Quote 42).

The prevailing climate may affect how an explosive odor presents to an EDC. Colder temperatures decrease the vapor pressure, decrease odor dissipation, keep molecules close to the source, potentially making initial detection more difficult but source identification easier. Warmer temperatures have the opposite effect and may degrade performance by making source identification more difficult. High humidity causes molecules to remain near the ground and may make initial detection more difficult but source identification easier. Low wind speeds in a consistent direction may enhance an EDC’s ability to identify odor while high wind speeds and inconsistent directions may degrade an EDC’s performance (Table 8, Quote 43). Finally, depressions in the terrain (e.g., tire ruts and drainage ditches) and obstructions in the physical environment (e.g., trees and elevated terrain) may change how odor moves and may create dead spaces that degrade performance unless directly investigated (Table 8, Quote 44).

## 4. Discussion

The purpose of this study was to explore the perspectives of subject matter experts on the factors that degrade the performance of EDCs. As this study revealed, many of these factors are best known by those who have observed them firsthand [13,14,18]. Learning from and documenting these accumulated observations unlocks operationally realistic performance assessment and future EDC research.

Searching for explosives is a prolonged and often repetitive process with extremely rare finds and lethal consequences for failure [19,20]. While the EDC team is currently the best mobile capability available to perform this task, they are pitted against intelligent adversaries and must perform at a consistently high level in challenging environments [5]. These realities underpin the importance of understanding, preparing for, and mitigating as much performance degradation as possible [8,21].

Performance degradation is to be expected when searching beyond a short duration. Each passing minute exposes the EDC team to more external stimuli, and the climate and effects of fatigue and boredom have a cumulative effect [19,22]. Some degrading factors (e.g., heat and humidity) are known before the search begins, have a relatively consistent effect, and manifest in externally observable signs [7,23,24]. Other factors are unknown beforehand (e.g., novel floor surface), may have an inconsistent effect (e.g., wind speed and direction), and may not result in changes in performance apparent to the handler (e.g., helicopter transport) [25]. Whatever form these factors may take, further exploration is needed to identify the specific impact on performance, develop monitoring tools, and devise mitigations to sustain performance wherever possible [26,27].

This expected degradation in performance highlights the critical role of the EDC handler [28,29,30]. This individual must anticipate alterations in performance or identify the subtle signs of early performance degradation. Conversely, the handler is the cause of numerous key effects including inadequate physical fitness, boredom (on both ends of the leash), and improper involvement in the search process [31,32,33,34]. Handlers require the training, experience, mentorship, and rapport with their EDC to be successful as much rests on the shoulders of these often junior personnel.

Every participant stressed the importance of preparation and acclimation to mitigate as much of the effect of these degrading factors as possible. Participants highlighted the importance of including all known factors in an EDC’s initial training and ensuring consistent access to continued exposure after initial certification. Selecting an innately confident future EDC is key as acclimation may mask behaviors that could emerge and degrade performance at inopportune times [9,35]. Some of the factors (e.g., elevated spaces and noxious stimuli from machinery) involve innate mammalian responses that may be difficult to fully overcome [36,37].

EDCs trained to perform criminal apprehension or assault functions face additional challenges. Some dogs may perform at equally high levels in both searching for explosives and searching for and engaging humans. Participants reported, however, the tendency for a dog to be better at one function than the other and the increased time and divided focus these utilizations require. Participants concluded that while financial pressures and organizational preferences drive the trend towards multiple utilizations, the best explosive detection is performed by single-purpose EDCs.

Finally, these results revealed that “operationally realistic” can mean many different things depending on how the EDC is utilized for a particular deployment. Organizations and researchers seeking to explore and assess EDC performance must carefully determine the specific type and degree of each factor the EDC in question will face.

The strengths of this study were the expert interview approach and the inclusion of highly experienced participants from diverse backgrounds. These factors resulted in documentation of institutional knowledge previously absent from the literature. Use of a qualitative approach limits our ability to generalize our findings to all EDC experts and all EDCs. However, the diverse experiences of our participants, the high degree of agreement in their answers and the detail with which they shared their knowledge has generated rich and detailed insights that can inform future research. This study only included U.S.-based participants, but these findings provide a starting point for future regional and comparative efforts.

Much future work is now necessary to prioritize and then explore the individual and cumulative effects of these factors. The specific type of effect, the onset, and the rate of impact needs to be determined for each factor. Where possible, tools should be developed to predict the effects of these factors before deployment and to identify their effects when they present during the deployment. Finally, performance should be supported by developing and implementing mitigations so the EDC team can do their lifesaving work with as little performance degradation as possible.

## 5. Conclusions

While the EDC team is capable of effective operational performance, the effectiveness of that team is under constant assault by a variety of external and internal factors. Expert EDC practitioners (handlers, trainers, and leaders) possess hard-earned tacit and explicit institutional knowledge critical to understanding these factors. The factors an EDC experiences differ according to their utilization, but many of the factors they will experience are known before the search commences. These elements must be adequately prepared for to ensure a sufficient level of operational performance is maintained. The factors revealed by these EDC practitioners should be incorporated into the selection, training, assessment, and utilization of EDCs. Future research can further illuminate individual factors and help develop mitigating strategies.

## Figures and Tables

**Table 1:** EDC utilization factor themes and exemplar quotations.

Interview Themes	Exemplar Quotations
Highly Controlled Operations	Quote 1. Yeah, we have the luxury of, and I say it, we’re a little bit bully-ish about it if you will. If we’re going to sweep something, we clear it prior to starting the sweep. And that’s for a couple reasons, not the least of which is safety for all people involved, even the handler, random people walking by, employees, customers, whatever. We’re going to clear those locations, or those vehicles, or the area prior to the actual sweep occurring. And we’re able to accomplish that with posted [personnel], police officers, [specialized personnel], that sort of thing.
Moderately Controlled Operations	Quote 2. Some of the environments, let’s say, for instance at (chain home improvement store). Not too long ago we had a bomb call there with one of my teams, and they had to search the whole parking lot which entailed probably 200–300 people, communicating, yelling, screaming around the area. On top of that they had cars with all forms of odors inside of them from narcotic odor to human odor to chemical odor, fertilizers.
Low Controlled Operations	Quote 3. You’re generally moving faster. There’s a lot of yelling. Once the breach team has executed its mission, everything gets loud. And there could be gunfire. There could be flashbangs. There could be all of these things as the team moves through, and those are all stimuli on the dog.
Criminal Apprehension or Assault Training or Utilization	Quote 4. Most of the law enforcement dogs have learned when they hear the siren that’s the, “Oh boy. I get to go get into some shit.” And it never fails, you’ll be trying to work, and you got four rookies that are still on their way there, so they come screaming up to the scene, with the siren still blasting, and at that point the dog’s ears perk up, he turns and looks at him and is like, “I want to go over that way, because obviously something good’s going on over there.”

**Table 2:** EDC team factors—handler behavior themes and exemplar quotations.

Interview Themes	Exemplar Quotations
Handler Behavior	Quote 5. For me, the handler affects the dog more than anything. You know a lot of times what I see is that these departments are getting handlers that want a dog just because it sounds cool to have a dog. And the handler education is the biggest for me. The more knowledge a handler has outside of the leash, starts understanding animal behavior, starts understanding all the different systems of training, and not just saying “Okay, I’m only holding a leash on a bomb dog,” the more success you’ll have as a team.
	Quote 6. If the handler isn’t enjoying the work or isn’t rewarding enough or appropriately, or let’s flip that coin, is punishing too aggressively. All of those things impact the mental welfare of the dog.
	Quote 7. Depending on the dog, the handler may be too involved. The dog may be fine, but the handler is too involved, depending on the history. If it’s a newer handler, newer dog, they haven’t learned each other, they haven’t associated whatever type of cues they have with each other, they don’t have a baseline, then that’s where things could get choppy.
	Quote 8. I call it the Groundhog Day effect. Where you continually have to do pre-sweeps at the same location multiple times, sometimes two, three times a week, and things like that. After a while there’s a malaise that sets in. The handler does not interact with the dog as frequently as he normally does. Dogs succumb to this easily. I’ve literally had to search something and 12 h later have to do it again. Like we maybe have a college game on Saturday and then once it was over, we’d have to come in and do the same thing again in less than 12 h later for the next day, Sunday, when the NFL came in and took over for their game. And then the human factor kicks in. Well, I just did this and now I’m doing it again. I know there’s nothing here. So I’m just going to hit the high points so to speak. And then get done with that and then a half hour later get that call out for a bomb threat where they actually found a precursor or something or an item that could be utilized in the manufacture of something. Not so much odor, but maybe batteries and wires. And then change how the handler feels about it, and then all of the sudden they become overly involved in their dog’s search.
	Quote 9. If the handler isn’t engaged and isn’t observant of the dog on where that dog is sniffing and if they’re actively sniffing, then that could ensure the dog’s either success or failure, depending on the particular situation. But that would be one factor. If the handler, not necessarily is lazy, just isn’t observant. So, I’ve talked about the lazy piece of it, but if they’re not observant, if they’re not experienced enough, don’t have that know-how to understand, to see their dog’s changed behavior.
Inappropriate Interaction with Handler	Quote 10. A lot of times for me what I see is dogs that are not successful lack the overall focus to task, focus to job, oftentimes lack of desire to search, or that that dog needs too much help from the handler. It’s not independent enough in its searching. That to me is a big sign if the handler always has to step in and help the dog.
	Quote 11. If he’s always fighting with the handler, you want him to go forward and he wants to go backward, you want him to go forward, and he goes left or right. That’s a dog that’s not working. It’s a dog that wants to do his own thing and it’s probably not something that we want in an explosives dog.

**Table 3:** EDC team factors—physical limitations of the EDC themes and exemplar quotations.

Interview Themes	Exemplar Quotations
Physical Limitations of the EDC	Quote 12. And then the dog has to be in good health. It’s got to be conditioned. If it’s not conditioned and in good health, you’ll get a product that will work okay for a short time, a very short time. Maybe 15 min, 20 min optimal. But after that, it will begin fading very quickly after that.
	Quote 13. Well case in point, I was watching this Lab working. I watched him go up high under a bunk bed to search, and I watched as the left rear foot rolled onto the back of the dog’s leg, and he stood on it while he was up and searching, which to me, was a telltale sign of a neurological issue, spondylosis or something like that. But then I noticed that he kind of rolled off of this search and every time he went high, he would go up and he’d immediately come back down but wouldn’t maintain it like a dog that was physically in good condition. Ultimately, we found out that’s what it was. There was actually a spondylosis issue. But the dog had a comfort issue that the handler wasn’t of that experience level that he knew that it was occurring.

**Table 4:** Manmade physical environmental factor themes and exemplar quotations.

Interview Themes	Exemplar Quotations
Manmade Surfaces	Quote 14. Anything that affects their footing. The sensation through the pads on their footing. Case in point is I’ve had to do some larger ships back in the day where I worked because we had a port. The grating on some of the decks and the steps provided a challenge because of the tactile feeling to the dog through their feet. There are some environments like that even the strongest dogs will sometimes shy away from.
Specific Locations	Quote 13. Basically, anything and everything is in play for that dog to deploy in. So, it’s just like basically any type of environment, that dog should be able to function in. I live in the mountains of (U.S. state). I’m looking at the side of a mountain right now. How many, actually outside of probably the military in Afghanistan back in the day in the mountainous regions can work a steep grade? And how many dogs that can’t work a large grassy area without taking a piss on something? Because they have to be able to identify odor can come from any environment, not just particular ones like cars. Like they strictly do cars. Some of these private companies, all they do is cars. And then I will hear them say he just does cars; he can’t do rooms. Why not? See what I’m saying? It is broad.
Confined Spaces	Quote 15. I would say one of the challenges is culverts, small types of tunnels. That is a challenge where it is one way down and it’s one way out. Did he go out, but you have to come backwards and there’s no room for turning around. Same thing with narrow passages in whatever building where it’s a long hallway, not much room. Same thing with aircraft, the restrooms in the aircraft, the cockpit.
	Quote 16. So again, if the training hasn’t happened or occurred for that dog to be in every single type of environment, lay down, and even though we typically teach the dog to sit for the response when they’re on odor or on source, if you teach the dog to lay down underneath an undercarriage and that’s their response because they just can’t sit, obviously, then the dog is going to fail if you don’t put them in those type of situations because the dog is not going to be comfortable in those type of scenarios. So, it’s all about getting the dog comfortable in certain environments.
Elevated Areas	Quote 17. Depending where you’re at, the elevation of something. That is a big factor. If you had to go search the top of something, the roof. Been there. That can affect the dog in the sense of what can be seen. In the sense of it’s glass, if you’re just high up on a high office and depending on the flooring, if it’s something that they could see down, let’s just say. This may be glass bottom like a patio or an offset of something where it’s enclosed with either glass or just your classic bars. The dog could see down and all of a sudden may show fear response.
Novel or Noxious Equipment	Quote 18. It was the top floors of, for instance, stadiums or big HVAC units where there is a bunch of HVAC equipment with the … what is it … all the ventilation, the pipes and all that in small, confined areas, kind of dark…
	Quote 19. You come around there and unintentionally you catch the exhaust of a generator or anything like that, that dog is going to shy back from that heat as soon as it hits his olfactory. That’s just automatically. It’s like putting your hand on the stove right when it’s been shut off. You’re going to pull back immediately, and dogs will do the same thing too.
Light	Quote 20. Yeah, working in low light because if you only work in light conditions, that’s what the dog is accustomed to. And then you work them in a dark environment, sometimes you’ll have a bit of a search drop off. It’s the transition and not being acclimated to it properly and making it more a part of their search environment.
Manmade Airflow	Quote 21. I would say in an environment where the air flow is uncontrollable. It could be a subway system. When a train pulls in, it pushes the air out. When a train leaves, it sucks the air with it. We’ve done a lot of stuff in mass transit, aviation, where the air flow in a terminal is just insanely brutal. Again, that’s a manmade structure, but because it’s climate controlled, a source might be in the right side of the building, but there’s no odor there because the odor’s pulling it to the left side of the building. You could be right on top of it and the air could be being sucked out. The bad guy could put his backpack near an intake of an air conditioning system. You put your nose right on it, no odors there because the air’s being sucked in through the ventilation system and it’s on the other side of the room.

**Table 5:** Natural physical environmental factor themes and exemplar quotations.

Interview Themes	Exemplar Quotations
Natural Surfaces	Quote 22. I think one that’s really tough for us is the one overseas in the desert. It was hard on the dogs as far as even the sand was hard on the dogs’ feet because it would splay their tendons of their paw out when they’re walking, which is not what they’re used to, the soil here in the United States, and they’d come up lame within a couple of days.
Subterranean Areas	Quote 23. Yeah, a novel environment might be the best way to capture it. I guess to give you an example is that we started trying to do some subterranean efforts and some of the dogs would struggle initially because they had never been in that type of environment.
Natural Airflow	Quote 24. Again, the surfaces, vegetation is a huge thing. When you’re doing landmines, it’s extremely difficult if you’re working in very tall vegetation because if you cannot see your dog, you can’t send them out, but also, you’ve got dead zones within the tall vegetation. It changes the wind current. A lot of the natural stuff, vegetation, terrain, ditches, you have dead zones. That almost got me in [African country]. They have the ponds. The vehicles would drive through and create divots and if you didn’t have your dog investigate it properly, you could miss landmines in that. I’ve seen that happen twice in [African country]. Like a pothole is enough to … Yeah, because it’s actually a dead zone within that pothole. You’ve got to ensure the dog actually sticks his head down into the lowest part of the pothole. A handler has to identify certain targets within that scope. If you’re doing road clearances, you do a lot of air scenting, you send your dog out, and you want your dog utilizing the wind, but you got to also be aware that when you come onto divots and cracks on the roads and stuff, those are dead zones. A lot of times you can actually have a dog physically go over and check within that crater or whatever.

**Table 6:** Climate environmental factor themes and exemplar quotations.

Interview Themes	Exemplar Quotations
Altered Handler Demeanor	Quote 25. The dogs don’t seem to do as bad as the people do, but I think the people affect the dogs. The dog’s hooked to the leash with the guy that’s basically not happy to be there, and that has an effect more so on the dog than the actual weather itself. I’ve been in situations where it was raining, and it was cold, and the dogs appeared to be cold as well, but a lot of times it’s that behavior that runs down the leash to the dog.
Effect of Heat and Humidity	Quote 26. No matter how upbeat the handler is and how ready to go the dog is, if it’s 130 degrees or even if it’s 89 degrees with 100% humidity, you can only go so long. Endurance goes way down, duration of workable time the dog can actively search goes way down. That high respiration, that inhalation, to keep that up real high, they need to be in very good shape, and I think no matter how good of shape they’re in, it gets so hot that it just saps the energy out of them. You can keep the dog moving, but in some of them, he’s just moving, he’s not searching anymore. The useful operational time or the useful search time of the dog goes way down, they need more breaks.
Requirement for Acclimation	Quote 27. You need to prepare the dogs for two to four weeks once you get the dogs into a hot environment, if you want to have them working optimally. And it doesn’t mean that you can’t work a dog right out of the box. You can take a dog from the United States that is on the East Coast and deploy that dog into Afghanistan or Iraq in 120-degree heat, you can put that dog immediately to work. But you’re not going to get optimal capability out of that dog if you don’t acclimate it first.
Novel Climates	Quote 28. The thing is you don’t know. You could take a dog and put them in the snow for the first time ever and the dog is so keen on working that they just work through those environmentals and just do their job. And, then you have another dog that comes out there that’s never seen snow and they’re eating the snow. It’s just too distracting. So, putting them in those situations ahead of time prepares you for the day that operationally it’s pouring rain, and someone goes, “We need you to go out and search those cars,” and you go, “Well, we have never searched in the rain.” You get your dog out in the rain and go to search, they’re probably going to look at you like, “I don’t know what you want me to do because we don’t do this.”

**Table 7:** Operational environmental factor themes and exemplar quotations.

Interview Themes	Exemplar Quotations
Repetition	Quote 29. The hardest part is keeping the dogs’ focus and motivation. It’s only going to be natural. “Dude, we’ve already run here before, there’s nothing here, there’s still nothing here.” If you repeatedly do the same thing and not present something new to the dog, a new environment to the dog even if you’re at the same place, you’re going to get less and less real focus to task unless you just have an absolute exceptional dog.
Other Animals	Quote 30. Our biggest problem was not the capability to detect explosive ordinance or explosive materials, it was the environment that allowed stray dogs to enter into the dog’s working environment. So now you’ve got a stray dog, and the dog sees a play buddy that he’s never seen before and gone. That was the one thing that drew that dog away instantly. And if we had a male dog, and that was a female dog in heat, gone. We lost all capability.
Personnel	Quote 31. External stimuli in the form of people is kind of a wildcard. When you’re around large crowds and specifically sporting events where there’s alcohol involved, people lose their good common sense. People can make eye contact with the dog and is that dog going to be aggressive, is it going to be skittish? Are they going to move in a manner that that dog feels threatened and distract the dog from the task at hand? Is the person handicapped and they wave their arms, or they walk differently than somebody else? The dog picks up on that anomaly.
Food Sources	Quote 32. For a lot of dogs, if there are easily accessible sources of food, that’s a problem. That is an instinct that’s hard to completely train away. As long as they are appropriately conditioned, I’m not so worried about the moving trucks or the moving people and the sounds and sights of that environment. The instinct to hunt, to eat, and to reproduce, those will be a challenge.
Sound	Quote 33. The raid environment is intense, noisy, often in the dark. So, there’s a lot of sensory things going on around the dog that can take the dog’s focus away from the mission at hand. Because they’re feeding off of us. We’re excited. We’re emotional. The dogs are reading that.
Transportation	Quote 34. A lot of departments are using air transport for the canine team. You’re physically taking that dog from point A to point B, dropping it in an environment with the expectation that that dog is going to be able to perform at a proficient level for an operational search. In my experience, about 50% of them wouldn’t find anything. They were just so stressed.
Criminal Apprehension or Assault Training or Utilization	Quote 35. If I’ve got a dual purpose dog out, I think the biggest thing to worry about is other people in the area, because since he has also learned it’s okay to bite people, and to watch bad guys, any time you put a strange person into his environment suddenly, he’s going to be overly suspicious and want to pay attention to that.
Anticipation	Quote 36. I’ve been on raids before where there’s no indication that there would be an explosive in there, but you have the dog in the event you run into something. So sometimes, the dog may not leave the kennel in the back of the HMMWV. But the dog’s as exhausted as everybody else is, just because of all the stimuli. But if you had to take the dog out, everybody is excited, and the dog is reading that. And the dog, depending on how long that raid took and where you are in the phase of the raid, might already be tired just because of the stimuli.

**Table 8:** Explosive odor environmental factor themes and exemplar quotations.

Interview Themes	Exemplar Quotations
Threshold	Quote 37. We do not train for operational relevance. We train for evaluation standards. If our canine community trained with operational hides, that produces a different scent picture, and it gives the dog a different threshold, we’d be much more effective at finding devices. We don’t know if we’re finding them, because we’re probably missing a lot. When’s the last time someone caught a bad guy with a quarter pound of black powder in a car door seam? Never. Now I get the need for search patterns, but why aren’t we training like that once these dogs graduate and they’re on the road for six months or a year? They should immediately go into threat devices. Put your training aids inside those pipe bombs, radios, human vests, laptops, put them in something because that’s what the bad guy’s going to do.
	Quote 38. They’re not going to put a block of C-4 in a vehicle door hinge or behind a headlight assembly. We continually do that, and it’s very concerning. It’s not just the dog that’s used to it. The handlers are used to seeing this massive change. As an evaluator, I’m 15 feet away, and I’m seeing dogs stop and change behavior real quick at source. Well, the handler doesn’t see that because they’re at four feet maybe six feet behind it. And a lot of these handlers are focusing on where they’re going next instead of watching their dog’s behaviors. Because we’re so in tune to evaluation standards. If we train to operational needs, we’ll blow evaluation standards away. But that’s not what we do. We’re backwards.
Concealment	Quote 39. I would say location is more difficult than amount. I think the dog would be able to find a small amount anywhere. I would rather there be a small amount than a bad guy trying to hide it efficiently.
Vapor Pressure	Quote 40. Certain odors in explosive detection you look for have lower vapor pressures, so sometimes even in an ideal environment it may be difficult for them to pick it up or pinpoint right away.
Set Time	Quote 41. The time the odor has been in place is important. Because even if it’s loosely packaged and easy to find, if they just dropped it wherever they dropped it, it’s not going to have had time to cook up and out. Originally very little if anything is coming out, but once it pushes up and out and gets up on top of the ground or out of the packaging, then the environment can get involved. So, the wind can push it around and make it more available to the dog.
Nature of Explosive	Quote 42. You don’t know if it’s going to be a homemade explosive, TATP or HMTD, [omitted country] C4, [omitted region] TNT. You can sort of try to train for it, but when you’re going into a lot of this stuff, you don’t know. When we were in [Middle Eastern country] doing searches and a lot of these landmines have been there for 30 years, so they from [omitted]. We didn’t have [omitted] mines to train on a lot. When we went into [Caucasian country], again, they were [omitted region], but then could have been a different type. You try to prepare, but you never can for sure know what you come across. We train on the homemade explosives, but they’re constantly changing mixes based on what’s available, what chemicals they can get, or they’re maybe actively using intelligence trying to defeat the dog.
Climate	Quote 43. If I always put out the training aids in nice, perfect climates and places, and the dogs will get used to that odor movement or finding the odor in a certain way. But, when it’s snowing, when it’s cold, when it’s raining, when it’s hot, when it’s humid, odor changes. In general, we see that it’s harder to find odor in cold temperatures than it is in warmer temperatures. But then if you throw high winds into the mix, it can create a lot of problems, and a miss.
	Quote 44. Environmental factors definitely affect odor, everything from temperature to humidity to wind to the terrain itself. If you’re in a tunnel or if you’re going along a mountainside, so hills, valleys, you name it. Is the dog going to be able to pick up the scent easily, or is it just going to go right over their head? Well, you’re going to have no luck based off of where the wind carries. If it’s heavily treed, will that end up blocking a lot versus an open field where the dog has the likelihood of picking up a scent?

## Data Availability

The raw data generated and analyzed during this study are not publicly available due to the sensitive nature of the data and ethics restrictions on data sharing. Respondents did not consent to have their data publicly shared.

## Supplementary Materials

The following are available online at https://www.mdpi.com/article/10.3390/ani11071978/s1, Interview Guide.
